# Prickly Heat

**Published:** 1910-03-26

**Authors:** 


### PRICKLY HEAT.

### Points in Treatment.

Prickly Heat, or Miliaria, may affect patients either in this country or in the tropics, but naturally it is an affection which comes out in hot weather or in hot climates rather than under temperate conditions. Occasionally, amongst those who live in humid districts in the tropics, it is exceedingly troublesome. The following prophylactic treatment against it has been recommended:

The individual should wear thin, light, woollen garments next the skin, expose the body to heat as little as possible, avoid constipation, and apply the following lotion locally:

| | |
|---|---|
| Acidi carbolici ... ... ... ... | ℥ss. |
| Acidi boracis ... ... ... ... ... | ℥j. |
| Zinci oxidi ... ... ... ... ... | ℥iss. |
| Glycerini ... ... ... ... ... | ℥ij. |
| Alcoholi ... ... ... ... ... | ℥ij. |
| Aquam q.s. ... ... ... ... ... | ad ℥vj. |

and a dusting powder consisting of—

Magnesii carb., acidi borici, pulv. amyli. aa ʒij.

When the entire body is involved, bran, starch, or alkaline baths are indicated. Hyde recommends:

| | |
|---|---|
| Acidi carbolici ... ... ... ... | ʒj. |
| Glycerini ... ... ... ... ... | ʒij. |
| Mentholis ... ... ... ... ... | ʒj. |
| Sp. vin. rectif. ... ... ... ... | ℥j. |
| Aquam q.s. ... ... ... ... ... | ad ℥viij. |

as a local application.

Pearse believes that in prickly heat the sebaceous glands are primarily at fault, and states that their own secretion causes an acute obstruction which then is secondarily maintained by the continued irritation of excessive perspiration. The disease is limited to those parts of the skin containing sebaceous follicles. His treatment consists in oily applications to the body-surface and the wearing of cotton next the skin.

